# An obstetric emergency called peripartum cardiomyopathy!

**DOI:** 10.4103/0974-2700.58664

**Published:** 2010

**Authors:** Nissar Shaikh

**Affiliations:** Department of Anesthesia/ICU and Pain Management, Hamad Medical Corporation, Doha-Qatar

**Keywords:** Heart failure, peripartum cardiomyopathy, postpartum, thromboembolism

## Abstract

Peripartum cardiomyopathy (PPCM) is a rare obstetric emergency affecting women in late pregnancy or up to five months of postpartum period. The etiology of PPCM is still not known. It has potentially devastating effects on mother and fetus if not treated early. The signs, symptoms and treatment of PPCM are similar to that of heart failure. Early diagnosis and proper management is the corner stone for better outcome of these patients. The only way to prevent PPCM is to avoid further pregnancies.

## INTRODUCTION

Peripartum cardiomyopathy (PPCM) is a rare dilated cardiomyopathy causing heart failure in women in late pregnancy or early postpartum. PPCM is rare in prepartum but 90% of the cases occur in first two months of postpartum period.[1] PPCM manifestations and treatment are the same as for heart failure but with consideration of effects of medication on the fetus.

PPCM is rare but causes significant morbidity and mortality in both mother and fetus; hence all clinicians and, in particular, acute care physicians should be aware of this disease. It was first described in the 18^th^ century but recognized as a separate clinical entity in 1930 and1971. Demakis *et al*. described criteria for the diagnosis of PPCM.[2] We will review PPCM in following sub-headings.

## DEFINITION

The definition of PPCM includes four criteria, three clinical and one echocardiographic:

1. PPCM occurs during last month of pregnancy or first five months after delivery.
2. Absence of an identifiable cause for cardiac failure.
3. Absence of heart disease prior to last five months of pregnancy.
4. Echocardiographic criteria - severe left ventricular systolic dysfunction, demonstrated by ejection fraction less than 45% or reduced shortening fraction of less than 30 %.[2]

## EPIDEMIOLOGY

The actual incidence of PPCM is not known. PPCM constitutes less then one per cent of all cardiovascular events related to pregnancy.[3] Incidences of PPCM in the United States are 0.03-0.06% of pregnancy. However, they are more common in Africa, 1:300 pregnancies. This may be due to the consumption of kanwa, a tradition, for 40 post-partum days. Kanwa is a dry salt and causes hypervolemia and hypertension. Ninety percent of PPCM occurs within two months of delivery.[4]

## RISK FACTORS

The following risk factors are associated with increased risk of PCCM:

Age over 30 years, pregnancy with multiple fetus,[5] multiparity, African descent,[6] maternal cocaine abuse,[7] long term tocolytic therapy[8] and familial. Selenium deficiency leading to PPCM is controversial.[9]

## ETIOLOGY

The exact etiology of PPCM is still not known, but the following hypothesis had been proposed:

(i) Familial; Familial clustering of PPCM is well known, it could be due to genetic or environmental factors.[10]

(ii) Myocarditis; Melvin proposed myocarditis as a cause for PPCM. Myocarditis could be viral or autoimmune, as with pregnancy there is increase susceptibility to both.[11] In one study endomyocardial biopsies in five patients showed features of myocarditis.

(iii) Abnormal immune response; the fetal cell enters maternal circulation and remains in circulation without rejection due to weak immunogenic paternal halotype of chorionic cell. If these cells lodge into the cardiac tissue it triggers a immune response.[12] Raised titers of immunoglobines and other autoantibodies in patients with PPCM are suggestive of abnormal immune response. However, contrast to it, Cenac *et al*. found no significant difference in levels of immunoglobines and other autoantibodies in PPCM and control group of patients (12).

(iv) Maladoption to stress of pregnancy; hyperdynamic circulation during pregnancy causes remodeling and transient hypertrophy of left ventricle, the exaggerated reduction in left ventricular systolic function with stress of gestational hypertension may contribute to heart failure in PPCM patients.[13]

## PATHOLOGY

Macroscopic findings are pale myocardium, dilated heart often with intramural thrombus in the ventricles. Endocardial thickening and pericardial fluid may be present. Non-specific findings are myocardial cellular hypertrophy and myofibril degeneration with areas of fibrosis and interstitial edema.[12] In a few patients it will show the feature of myocarditis with the presence of inflammatory cell infiltration of myocardium, focal necrosis, variable hypertrophy and fibrosis of the myocardium.

## CLINICAL PRESENTATIONS

Some of the most common presentations of PPCM are dyspnoea, cough, orthopnea, paroxysmal nocturnal dyspnoea, which may be confused initially due to similar presentation in late pregnancy. The patient may have arrhythmia, rarely, PPCM can present with thromboembolic manifestations. PPCM is manifested after 38 weeks of pregnancy when chronic heart disease pregnant patients develops sign and symptoms of heart failure in the second trimester of pregnancy due to stress of hemodynamic overload.

## DIAGNOSIS

PPCM patients present with dyspnoea and other signs and symptoms of left heart failure. The physical examination and x-ray chest may reveal cardiomegaly and pulmonary edema. Although the x-ray chest is not essential to diagnose PPCM, its routine use during pregnancy should be discouraged. The electrocardiogram (ECG) may be normal or show sinus tachycardia, atrial fibrillation or nonspecific ST segment changes.

The echocardiography will rule out other valvular diseases and at the same time diagnose reduction in the left ventricular ejection fraction and dilatation of cardiac chambers.[14] Most of the cardiologists would consider PPCM, if left ventricular ejection fraction less than 50% and in the presence of other two previously mentioned criteria for diagnosis of PPCM. Other nonspecific echocardiography findings in PPCM are left atrial enlargement, mitral regurgitation and small pericardial effusion. The endomyocardial biopsy may show features of myocarditis, but the decision for biopsy should be taken after thorough discussion between patient and treating physicians. Viral and bacterial culture as well as coxasckie B titer should be considered in selected cases. Invasive hemodynamic monitoring will show elevated right and left heart filling pressures with low cardiac index.

## DIFFERENTIAL DIAGNOSIS

PPCM should be differentiated from other forms of cardiomyopathy, heart failure, pulmonary thromboembolism, severe eclampsia and pneumonia. From history, physical examination and investigations one must exclude, myocardial infaction, idiopathic dilated cardiomyopathy and valvular heart disease.[8]

## MEDICAL MANAGEMENT

Management of PPCM is similar to other types of heart failure, apart from concerning the adverse effect of treatment on fetus or breast-feeding infant. The aim of therapy in PPCM is to reduce preload, after load and increase the cardiac contractility. Heart failure during pregnancy can be acute or acute on chronic. Pregnant patients with known cardiovascular disease can present in stable condition in early stages of pregnancy; their management is mainly adjustment of their cardiac medications and regular monitoring for cardiac decomposition, an initial detailed cardiovascular and careful physical examination should be done, New York heart association functional status should be documented, ECG and echocardiogram should be performed. Patients presenting in decompensate cardiac status during pregnancy or in the peripartum period, may be known to have cardiac disease or it may be acquired during the pregnancy such as peripartum cardiomyopathy. Management of these patients includes detail history and physical examination, evaluation of severity of decompensation. An ECG may reveal deteriorating left ventricular functions, arrythmia, LVH or arterial abnormality. The aims of therapy in these patients include optimizing hemodynamics, reducting after load, optimizing preload and cardiac contractility. These can be achieved by treatment of pulmonary congestion, control of hyper/hypotension, treatment of cardiac arrytheamia and prevention of thromboembolic events. Digoxin is safe to use in pregnancy. Diuretics can be used if salt restriction is not sufficient. Beta-blocker improves left ventricular functions in patients of PPCM, but ACE inhibitors are the drug of choice in postpartum PPCM.

Ventricular arrhythmias should be treated aggressively in cases of PPCM. Class III antiarrythmic medication is the best option.[15] Intravenous medications are needed in PPCM patients admitted to the intensive care therapy unit. Therapy with ionotropes such as dobutamine, adrenaline and milrinone, should be directed by invasive cardiac monitoring. While interpreting the invasive hemodynamic monitoring, one should take into account of normal changes that occurs during pregnancy.[12] The immunosuppressive therapy to be started after two weeks of standard therapy with biopsy proven myocarditis, but still efficacy is unclear.[16]

Anticoagulation in PPCM is a must as pregnancy itself is a hypercoagulable state, in addition to PPCM, dilatation of heart and turbulent flow of blood.[17] Before delivery, unfraction or low molecular weight heparin is the choice, while in postpartum period warfarin is used.[18] Cardiothoracic medication, effects on fetus and their secretion in the breast milk are shown in detail in Table 1. Occasionally, when medical therapy fails in patients with PPCM, need for mechanical cardiovascular support (intra-aortic balloon pump, ventricular assisting device) or even cardiac transplant has been reported in the literature.[19]

**Table 1:** Medications and their effect on fetus and their secretion in breast milk

Medication	Potential side-effects	Safety in pregnancy	Safety during breast feeding
ACE inhibitors	Embroyopathy	Not	Yes
AR blocker	Embroyopathy	Not	Yes
B-blockers	Fetal bradycardia/ IUGR	Yes	Yes
Hydralizin	None	Yes	Yes
Digoxin	Low birth weight	Yes	Yes
Diuretics	Reduction in uteroplacental perfusion	Unclear	Yes
Nitrates	Fetal distress with maternal hypotension	Yes	No data
Lidocain	Fetal CNS depression	Yes	Yes
Procainamide	Maternal osteoporosis	Yes	Yes
LMWH	Hemorrhage	Yes	Yes
Heparin	Hemorrhage/maternal osteoporosis/thrombocytopenia	Yes	Yes
Warfarin	Warfarin embroyopathy	Yes after 12 weeks	Yes
Adenosine	Non reported	Yes	No data

ACE INHIBITORS: ANGIOTENSIN CONVERTING ENZYME INHIBITORS, AR BLOCKER: ANGIORECIPTOR BLOCKER, IUGR: INTRA-UTERINE GROWTH RETARDATION, CNS: CENTRAL NERVOUS SYSTEM

There is no indication to terminate pregnancy as a form of therapy. If concurrent pre-eclampsia, maternal hemodynamic are inadequate to support fetus or therapy to support mother puts fetus at risk, early delivery of near term fetus is strongly recommended.[20]

## COMPLICATIONS

The most common complication is thromboembolism.[2] A premature delivery rate of 25% has been reported in cases with PPCM.[20] PPCM cases had increased incidence of cesarean section up to 40%.

## PROGNOSIS

Maternal outcome; the mortality in mother who develops PPCM is 15-50%, various factors such as, black women, multiparity, LVEF less than 30% are indicators of worst maternal outcome.[2] Neonatal outcome; preterm birth occurs in up to 25% of patients, a few intra-uterine fetal deaths and 40% of patients with PPCM under go cesarean section for obstetric reasons.[4]

Predictors of persistent left ventricular dysfunction are, LVEF less than or equal to 30%, fractional shortening less than or equal to 20% and left ventricular end diastolic dimension greater than or equal to six cm.

## PREVENTION

In patients who have recovered from left ventricular failure due to PPCM there is a high risk of PPCM in subsequent pregnancy and so the best way to avoid PPCM is avoid subsequent pregnancy.[21]

## CONCLUSION

Peripartum cardiomyopathy (PPCM) is a rare pregnancy-induced dilated cardiomyopathy, more common in the post-partum period. The exact etiology of PPCM is not known. Various hypothesis have been proposed. The incidence of PPCM varies with geographical variation. Risk factors for PPCM are elder pregnancy, multiparity, African descent, pregnancy with multiple fetus, maternal cocaine abuse and longer oral tocolytic therapy.

Clinical presentations are dyspnea, palpitations, rarely present as thromboembolic phenomenon. Echocardiography is a must for the diagnosis of PPCM as it will show reduction in ejection fraction and rule out other cardiac lesions.

Management of PPCM includes optimizing hemodynamics, reduction in after load, optimizing preload and cardiac contractility. These can be achieved by treatment of pulmonary congestion, control of hyper/hypotension, treatment of cardiac arrytheamia and prevention of thromboembolic events. The thromboembolic phenomenon is the most feared complication of PPCM. The reported mortality of PPCM is 15-50%. The best prevention of PPCM is to avoid subsequent pregnancies.
